# Underrepresented Minority High School and College Students Report STEM-Pipeline Sustaining Gains After Participating in the Loma Linda University Summer Health Disparities Research Program

**DOI:** 10.1371/journal.pone.0108497

**Published:** 2014-09-24

**Authors:** Lorena M. Salto, Matt L. Riggs, Daisy Delgado De Leon, Carlos A. Casiano, Marino De Leon

**Affiliations:** 1 Center for Health Disparities and Molecular Medicine, School of Medicine, Loma Linda University, Loma Linda, California, United States of America; 2 Department of Psychology, California State University San Bernardino, San Bernardino, California, United States of America; University of North Carolina at Chapel Hill, United States of America

## Abstract

An urgent need exists for graduate and professional schools to establish evidence-based STEM (science, technology, engineering, and math) pipeline programs to increase the diversity of the biomedical workforce. An untapped yet promising pool of willing participants are capable high school students that have a strong STEM interest but may lack the skills and the guided mentoring needed to succeed in competitive STEM fields. This study evaluates and compares the impact of the Loma Linda University (LLU) Summer Health Disparities Research Program on high school (HS) and undergraduate (UG) student participants. The primary focus of our summer research experience (SRE) is to enhance the research self-efficacy of the participants by actively involving them in a research project and by providing the students with personalized mentoring and targeted career development activities, including education on health disparities. The results of our study show that our SRE influenced terminal degree intent and increased participant willingness to incorporate research into future careers for both the HS and the UG groups. The quantitative data shows that both the HS and the UG participants reported large, statistically significant gains in self-assessed research skills and research self-efficacy. Both participant groups identified the hands-on research and the mentor experience as the most valuable aspects of our SRE and reported increased science skills, increased confidence in science ability and increased motivation and affirmation to pursue a science career. The follow-up data indicates that 67% of the HS participants and 90% of the UG participants graduated from college with a STEM degree; for those who enrolled in graduate education, 61% and 43% enrolled in LLU, respectively. We conclude that structured SREs can be highly effective STEM strengthening interventions for both UG and HS students and may be a way to measurably increase institutional and biomedical workforce diversity.

## Introduction

In 2010 the original committee members of the “Rising Above the Gathering Storm” report revisited the recommendations, policies enacted and progress since the publication of this report first questioned the science, engineering and thereby economic and global competitiveness of our nation [1]. The members unanimously agreed that despite some legislative support and scattered victories along the way, the nation’s outlook had worsened in the five-year span. The committee recognized the lagging state of the U.S. K-12 education as an underlying concern and concluded that innovation that draws on a diverse and competent science and engineering national workforce will be critical for the continued prosperity of the nation [1]. The goal of sustaining and strengthening the science and engineering workforce resonated with lawmakers who further mandated a study on broadening the participation of all Americans, including minorities that are underrepresented in science and engineering [2].

As of 2010, African Americans, Hispanics, Native Americans, and Native Hawaiian and Pacific Islanders together comprised 31.1% of the total U.S. population (U.S. Census Bureau: www.census.gov). Collectively, however, these underrepresented minorities (URMs) earned only 13.1% of all STEM (science, technology, engineering and math) research doctorates in that same year according to data from the Survey of Earned Doctorates [3]. The glaring lack of diversity in the biomedical research enterprise prompted NIH to review all of its training programs and seek new initiatives that will address this serious national challenge [4]. If population projections prove true, the overall minority population will become the majority population by the year 2050 [5], and the current underrepresentation trends in STEM fields will continue or worsen unless aggressive measures to prepare, recruit and retain URMs are adopted.

Interventions aimed at strengthening the STEM academic pipeline have prioritized undergraduates and have focused on the undergraduate to graduate level leakage point [2]. With some notable exceptions, these measures have had limited success in propelling an adequate number of URMs into graduate and professional schools, especially given the dearth of URM college graduates with STEM degrees despite comparable STEM interest rates reported for incoming freshmen [6], [7]. Rather, the considerable leakage of URMs after the first and second-year undergraduate gateway science courses and the longer time-to-degree completion rates for STEM compared to non-STEM majors indicate a need for earlier, pre-college interventions [6], [8], [9]. Although a rigorous math and science high school curriculum is fundamental, as Seymour and Hewitt point out [10], even talented students leave the sciences, making pre-college programs that capitalize on STEM interest and bolster science skills crucial for a successful science career trajectory [10]–[12].

A summer research experience in high school can develop science self-efficacy, improve science process skills and begin a lifelong commitment to inquiry-based learning that may subsequently stem the tide of attrition before the freshman year in college occurs [2], [13], [14]. While most of the published literature has addressed the utility of research experiences at the undergraduate level, some published results on high school research experiences are emerging [15]–[18]. In this article, we evaluate and compare the impact of the Loma Linda University (LLU) Summer Health Disparities Research Program on high school and undergraduate student participants. From the start, the inclusion of local high school students in our summer research program has been central to our goal of strengthening a STEM pipeline of underrepresented minorities that would increase the student diversity at our institution and nationwide. The primary focus of the program is to enhance the research self-efficacy of the participants by actively involving them in a summer research project and by providing the students with personalized mentoring and targeted career development activities, including education on health disparities.

## Methods

### History of the Apprenticeship Bridge to College (ABC) Program

The Apprenticeship Bridge to College (ABC) summer research experience is an 8-week research internship program for high school students in the Inland Empire region of Southern California. This high school research experience was first known as RAMP (Research Apprenticeship for Minorities Program) from 1997–1999, then pre-URSP (pre-Undergraduate Research Scholars Program) from 2001–2003, after that UPWARD from 2004–2005, and finally renamed the Apprenticeship Bridge to College (ABC) program from 2006 onward. Since its inception in 1997, the ABC program has awarded 163 internships to 132 participants, 28 of which have participated in the program more than once and 16 of which have participated in both the Undergraduate Training Program (UTP) and the ABC program (for multiple summer participants, the follow-up outcomes are counted only once and only for the ABC program). The ABC program has had the same uninterrupted leadership oversight since 1997. Currently, the ABC program is the only structured, university-based research training program in the Inland Empire that targets high school students.

### ABC Eligibility and Selection Criteria

The ABC program provides an average of 15 paid research internships per summer to underrepresented minority students who aspire to become biomedical scientists or physicians and are committed to serving their community. In order to be considered for admission, the high school students must possess a 3.0 minimum grade point average (GPA) and submit a completed application with a personal statement and 2–3 letters of recommendation from math or science teachers and/or school counselors. Exceptions to the GPA requirement have been considered and granted under special circumstances. A key component in the success of the program is our longstanding partnership with local schools, teachers, counselors, school principals and superintendents. All applicants are currently invited to the LLU campus for the “ABC Invitational Night” where the students and their parents are given the opportunity to meet the leaders and faculty of the program, learn more about the summer research experience and listen to a keynote address by the Dean of the School of Medicine on the importance of biomedical research. Each high school applicant is interviewed twice by faculty members and basic science doctoral degree students. The high school students are evaluated on academic performance, interest in the biomedical sciences, letters of recommendation, personal statement, community service record, and performance in the interview. Finally, the admissions committee selects the ABC participants based on their overall application and the interview rankings.

### History of the Undergraduate Training Program (UTP)

The goal of the UTP is to engage underrepresented minority students in biomedical research in order to increase the number of biomedical scientists and physicians committed to addressing health disparities. The UTP summer research experience is an 8-week hands-on program for college students that are enrolled full time in local and nationwide colleges and universities. The UTP runs simultaneously and side-by-side with the ABC program, and both groups of trainees participate in the skill building and social activity components to allow for ample peer mentoring opportunities. The majority of the UTP participants come from out-of-state colleges and universities and reside in the LLU campus dormitories for the duration of the program. This undergraduate research experience was first known as the Undergraduate Research Scholars Program (URSP) from 2001–2005 and renamed the Undergraduate Training Program (UTP) from 2006 onward to reflect the new mission of the Center for Health Disparities and Molecular Medicine. From 2001 through 2012, a total of 179 summer research internships were awarded to 139 UTP participants, 13 of which participated in the program more than once. The UTP has also had the same uninterrupted leadership oversight since its start in 2001.

### UTP Eligibility and Selection Criteria

The eligibility and selection criteria for UTP participants are similar to that of the ABC program. An average of 15 paid summer research internships are awarded to promising underrepresented minority students at the undergraduate level who aspire to become biomedical scientists or physicians committed to serving their community. In order to be considered for admission, the college students must possess a 3.0 minimum GPA and submit a completed application with a personal statement and 2–3 letters of recommendation from math or science teachers. Most UTP applicants are already STEM majors, and many are considering graduate education at the time of application. Unlike the ABC program applicants, most UTP applicants are not interviewed in person. Therefore, the student application files are carefully reviewed by the admissions committee for evidence of community service involvement and a willingness to engage in biomedical research related to health disparities.

### Summer Research Experience Program Components

Consistent with Bandura’s general theory of self-efficacy beliefs [19], [20], the intervention described hereafter increases research-specific self-efficacy by enabling enactive mastery experiences through a hands-on research internship that includes doing, interpreting, and presenting research under the direct supervision of productive research mentors. The participants are matched with individual mentors based on their application-stated research interest. All the students are required to participate in an orientation process that introduces them to the program leaders, their respective faculty mentors, peer participants, and the laboratory and summer research program expectations. During orientation the participants also complete all the required employment documentation and are given a tour of the campus and the research and clinical facilities.

Our “research-apprenticeship” internship immerses students in a rigorous basic science lab or public health research experience where for 8 weeks they become part of a lab and perform research under the guidance of a research mentor. The students devote 40 hours per week to hands-on research and program activities and are placed in laboratories to interact closely with more senior trainees in the academic pipeline and benefit from cross-mentoring in a successful research environment. The summer participants and their respective research mentors discuss and decide on an individual project, define goals and experiments, and adopt a reasonable timeline for completion. The goal of the program is for participants to achieve salient vicarious experiences as ethnically diverse mentors, presenters, and program leaders enable them to identify with scientists already succeeding and contributing to STEM research. Verbal persuasion also comes from those same sources, and constructive support is provided consistently in labs, seminars and workshops. It is also anticipated that the time spent in a functioning lab on an active research campus will reduce the “mystery” and intimidation factor often associated with STEM fields of research, thus reducing physiological and affective states that can reduce motivation to persist toward a desirable goal.

Beyond their own summer research project, the participants also complete supplemental group learning activities or modules that teach responsible research conduct, how to conduct scientific literature searches, effective oral presentations, scientific poster presentations, careers in biomedical and health disparities research and the art of networking. Although health disparities research is incorporated throughout the 8-week long internship, another major component of the summer research program is the weekly health disparities education curriculum. Specific to the ABC program is the Verbal, Analytical Reading and Writing Skills module, in which the students are asked to describe their lab work and take back to their own research what they learn in this reading and writing module. Although the UTP participants do not attend a specific Verbal, Analytical Reading and Writing Skills module, they are incorporated into journal club meetings within their laboratories. The culminating summer research experience event is the Annual Health Disparities Research Symposium where the participants are required to write a 1-page abstract, create a poster and give a formal poster presentation on their individual research project.

### Data Sources

The data used for this study was collected as part of the ongoing evaluation for the Summer Health Disparities Research Program and was analyzed retrospectively for the 1997–1999 and 2001–2012 program years. The LLU Institutional Review Board (IRB) determined that this study was exempt from IRB approval as outlined in the federal regulations for the protection of human subjects, 45 CFR Part 46.101(b). A waiver of informed consent for research was approved for this study because the research risks would be minimal and would not adversely affect the rights and welfare of the participants. The demographic, school enrollment, terminal degree intent, and research career intent data were drawn from the program applications and the follow-up files. Data sources for age and GPA were verified using employee files and official application transcripts, respectively. After completion of the program, the participants were followed-up through phone calls and emails. When possible, enrollment, graduation and posted degree for both undergraduate and graduate education were tracked through the National Student Clearinghouse (www.studentclearinghouse.org). At intermittent time points since 2006, the ABC and UTP participant groups have completed evaluation surveys before (PRE) and after (POST) the summer research experience. These evaluations ask the participants to rate themselves on a variety of research-related skills and also contain 10 academic and 10 research self-efficacy Likert-items. In particular, the exit survey (POST) contains additional free-answer questions that probe for participant reactions to the summer research experience and changes, if any, in future science or research career plans.

### Statistical Analysis

The statistical analyses were performed using IBM SPSS Statistics Software, version 22 (Armonk, New York). The data is shown for the high school (ABC) and the undergraduate program (UTP) separately and for both programs combined. The frequencies for the qualitative characteristics are shown as percentages based on valid data and excluding any missing data. The descriptive characteristics are shown as means ± SD or means ± SEM, as indicated. Pearson’s chi-square statistic was used to test for proportional differences in intention to incorporate research into future careers before the summer research experience by group and McNemar’s chi-square statistic was used to test changes in that same proportion before (as specified on the program applications) and after the summer research experience (as specified on the exit surveys). The General Linear Model (GLM) was used to compare research skill self-ratings and the summated academic and research self-efficacy values before and after the summer research experience. A repeated measures model that included a two-level (PRE and POST) within-subjects factor was used for each independent group; consequently, an eta squared (η^2^ = Sum of Squares_Effect_/Sum of Squares_Total_) effect size estimate is reported for each of the research skill and self-efficacy outcomes. The interpretation of the reported effect size was according to Cohen’s effect size magnitude criteria for the behavioral sciences (η^2^ = 0.01 small; 0.09 medium; 0.25 large) [21]. The academic and research self-efficacy summated variables are based on 10 academic and 10 research self-efficacy Likert items, respectively. A Cronbach’s α coefficient of 0.732 for the academic self-efficacy scale items and a Cronbach’s α coefficient of 0.770 for the research self-efficacy scale items indicate adequate reliability. Pearson’s correlation coefficient was used to determine the linear relationship between the summated academic and research self-efficacy variables. Finally, binary logistic regression was used to estimate the likelihood of pursuing a biomedical doctoral degree (not likely/very likely) after the summer research experience. Type 1 error was set at α = 0.05 for statistical significance.

### Content Analysis

The free-answer responses on the summer research experience exit surveys were reviewed for content analysis. The ABC and the UTP participants were asked to provide details regarding the most valuable or most beneficial aspect of the summer research experience and were also asked about the impact of the experience on future goals with respect to school and/or career. The participant responses to those open-ended question stems were coded for conceptual analysis; each participant response was coded into a category through selective reduction and each participant response was only counted once for each question stem. In rare instances when the participants identified two distinct concepts, the first item written by the participant was used as the classifying concept in order to maintain a “most important” ranked concept list. None of the participants listed more than two distinct concepts for either of the question stems. To see if the 4 major sources of self-efficacy information were contributing to the participant-perceived changes after the summer research experience, the responses regarding the most valuable/most beneficial aspect of the summer research experience were further categorized under mastery experiences, vicarious experiences, verbal persuasion, and physiological and affective states in accordance with Albert Bandura’s self-efficacy theory [20].

## Results

### Participant Demographic Characteristics

The demographic characteristics of the students at the time of participation are summarized in Table 1. For both the ABC and UTP programs, the majority of the participants were females. The average age of the ABC program participants was 16.5 years while the average age of the UTP participants was 20.1 years. Most ABC program students participated right after their junior and senior year of high school whereas the UTP participants primarily participated after their sophomore and junior year of college. As stated on our website and on our printed recruitment materials, the high school students were selected for participation in great part based on their willingness to pursue a biomedical research career, and, therefore, a great majority (93%) of the sampled participants aspired to major in a STEM discipline. The great majority of the selected UTP participants (90%) were STEM majors at the time of selection.

**Table 1:** Participant Demographic Characteristics at the Time of the Summer Research Experience, ABC (1997–1999, 2001–2012) & UTP (2001–2012)*.

	ABC	UTP	ABC & UTP
Number of Participants	132	139	271
Male, % (n)	31% (39 of 127)	27% (37 of 139)	29% (76 of 266)
Female, % (n)	69% (88 of 127)	73% (102 of 139)	71% (190 of 266)
Age^a^, Years (Mean ± SD)	16.5±0.9	20.1±2.6	18.6±2.7
GPA^a^ (Mean ± SD)	3.78±0.2	3.62±0.3	-
Grade Level^a^, % (n)			
Freshmen	3% (3 of 103)	13% (18 of 139)	9% (21 of 242)
Sophomore	14% (14 of 103)	43% (60 of 139)	31% (74 of 242)
Junior	55% (57 of 103)	37% (52 of 139)	45% (109 of 242)
Senior	28% (29 of 103)	7% (9 of 139)	16% (38 of 242)
Aspired to Major in aSTEM Discipline^a^, % (n)	93% (71 of 76)	-	-
STEM Declared Major^a^, % (n)	-	90% (125 of 139)	-
High School Type, % (n)			
Private	25% (28 of 113)	-	-
Public	75% (85 of 113)	-	-
Attended SED-populationServing High School^b^, % (n)	71% (60 of 85)	-	-
Attended Minority-servingCollege/University^c^, % (n)	-	85% (118 of 139)	
UnderrepresentedMinority (URM)^d^, % (n)	81% (83 of 103)	96% (134 of 139)	90% (217 of 242)
Self-reported Ethnicity, % (n)			
African American/Black	22% (23 of 103)	53% (74 of 139)	40% (97 of 242)
Asian	13% (13 of 103)	3% (4 of 139)	7% (17 of 242)
Hispanic/Latino	56% (58 of 103)	41% (57 of 139)	48% (115 of 242)
Native American/American Indian	1% (1 of 103)	2% (3 of 139)	2% (4 of 242)
Non-HispanicWhite/Caucasian	7% (7 of 103)	1% (1 of 139)	3% (8 of 242)
Other/Did not specify	1% (1 of 103)	-	0.4% (1 of 242)

ABC = Apprenticeship Bridge to College, UTP = Undergraduate Training Program, GPA = Grade Point Average, STEM = Science, Technology, Engineering, Math.

*Percentages were calculated based on valid data (excluding missing data) and may not add to one-hundred due to rounding.

a As indicated on program application and just prior to participation.

b For our purposes, a socioeconomically disadvantaged (SED)-population serving school included CA public high schools where the socioeconomically disadvantaged student population percent ≥50% and/or the high school academic performance index (API) <7.0 (www.cde.ca.gov/ta/ac/ap/).

c Participant’s college/university was designated an accredited postsecondary Minority Institution by the U.S. Department of Education and/or designated a Hispanic Serving/High Hispanic Enrollment Institution or designated a Historically Black College/University.

d Current NIH Biomedical Research Training Inclusion Criteria for Underrepresented Minorities (URM) includes African Americans/Blacks, Hispanics/Latinos, Native Americans/American Indians and Pacific Islanders.

For the ABC participants that attended public high schools, 71% attended a socioeconomically disadvantaged (SED) population-serving high school where the SED student population was greater than or equal to 50% and/or the California high school academic performance index (API) was less than 7 (www.cde.ca.gov/ta/ac/ap/). At the time of participation, roughly 85% of the UTP participants were enrolled in a minority-serving college or university, as designated by the U.S. Department of Education. When both groups are combined, about 90% of the participants were considered underrepresented minorities as currently defined by the National Institutes of Health training inclusion criteria (http://grants.nih.gov/training/faq_diversity.htm).

### Terminal Degree and Research Career Intent

To gauge changes in terminal degree and research career intent before and after the summer research experience, intent for each student was taken from the program applications and compared to what was indicated on the exit surveys. The results are shown in Table 2 for a subgroup of ABC (*N* = 53) and UTP (*N* = 38) participants for whom we have valid paired-data. For the most part, ABC participants varied more than the UTP participants with regard to their terminal degree intent before the summer research experience; still, about 66% of them indicated an MD as their intended terminal degree before the summer research experience. At the end of the summer research experience, the ABC participants increased in intent for MD/PhD’s and PhD/DrPH’s in public health. The UTP participant subgroup showed less variability in terminal degree intent before the summer research experience, yet almost twice as many UTP participants were likely to indicate intent for an MD/PhD degree after the summer research experience.

**Table 2:** Participants’ Terminal Degree and Research Career Intent Before and After the Summer Research Experience, ABC & UTP Participants (2001–2012)*.

	ABC (*N* = 53)		UTP (*N* = 38)		ABC & UTP (*N* = 91)	
	PRE, % (*N*)	POST, % (*N*)	PRE, % (*N*)	POST, % (*N*)	PRE, % (*N*)	POST, % (*N*)
**Terminal Degree Intent**						
MD	66% (35 of 53)	30% (16 of 53)	40% (15 of 38)	21% (8 of 38)	55% (50 of 91)	26% (24 of 91)
MD/PhD	4% (2 of 53)	43% (23 of 53)	26% (10 of 38)	45% (17 of 38)	13% (12 of 91)	44% (40 of 91)
PhD-Basic Science	15% (8 of 53)	17% (9 of 53)	13% (5 of 38)	16% (6 of 38)	14% (13 of 91)	17% (15 of 91)
PhD-Behavioral Science	4% (2 of 53)	2% (1 of 53)	5% (2 of 38)	3% (1 of 38)	4% (4 of 91)	2% (2 of 91)
PhD/DrPH-Public Health	2% (1 of 53)	6% (3 of 53)	-	-	1% (1 of 91)	3% (3 of 91)
PharmD	-	-	11% (4 of 38)	8% (3 of 38)	4% (4 of 91)	3% (3 of 91)
DDS	-	-	5% (2 of 38)	5% (2 of 38)	2% (2 of 91)	2% (2 of 91)
Allied Health Professional^a^	6% (3 of 53)	-	-	-	3% (3 of 91)	-
DVM	-	-	-	3% (1 of 38)	-	1% (1 of 91)
Master’s	2% (1 of 53)	2% (1 of 53)	-	-	1% (1 of 91)	1% (1 of 91)
Nursing	2% (1 of 53)	-	-	-	1% (1 of 91)	-
**Research as Part of Career Intent**						
Yes	23% (12 of 53)^b^	87% (46 of 53)	47% (18 of 38)^b^	92% (35 of 38)	33% (30 of 91)	89% (81 of 91)
No	77% (41 of 53)^b^	13% (7 of 53)	53% (20 of 38)^b^	8% (3 of 38)	67% (61 of 91)	11% (10 of 91)
p-value^c^	p<0.001**		p<0.001**		p<0.001**	

ABC = Apprenticeship Bridge to College, UTP = Undergraduate Training Program.

*Percentages are calculated using valid paired data and may not add to one-hundred due to rounding; PRE data is from participant’s program application and prior to participation in the summer research experience, POST data is from the summer research experience exit survey.

a Allied health professional category includes Physical Therapy, Occupational Therapy and Optometry.

b Before the summer research experience, more UTP participants indicated an intent to incorporate research into their future careers as tested by Pearson’s chi- square (*χ* ^2^(1, *N* = 91) = 6.12, *p* = 0.013).

c P-value shown is based on McNemar’s chi-square statistic for changes in proportions in a binary variable over time, **(*χ* ^2^(1, *N* = 91) = 2.68, *p*<0.001).

Before the summer research experience, more UTP participants (47%) than ABC participants (23%) indicated an intent to incorporate research into their future careers (*χ* ^2^(1, *N* = 91) = 6.12, *p* = 0.013). Interestingly, after the summer research experience, the ABC participants indicated a greater increase (+64%) in intent compared to the UTP participants (+45%). Of particular significance, when combined and after the summer research experience, the proportion of ABC and UTP participants that indicated an intent to incorporate research into their future careers increased dramatically to 89% of the sample, and this change was statistically significant (*χ* ^2^(1, *N* = 91) = 2.68, *p*<0.001).

### Research Skills

**Figure 1:**
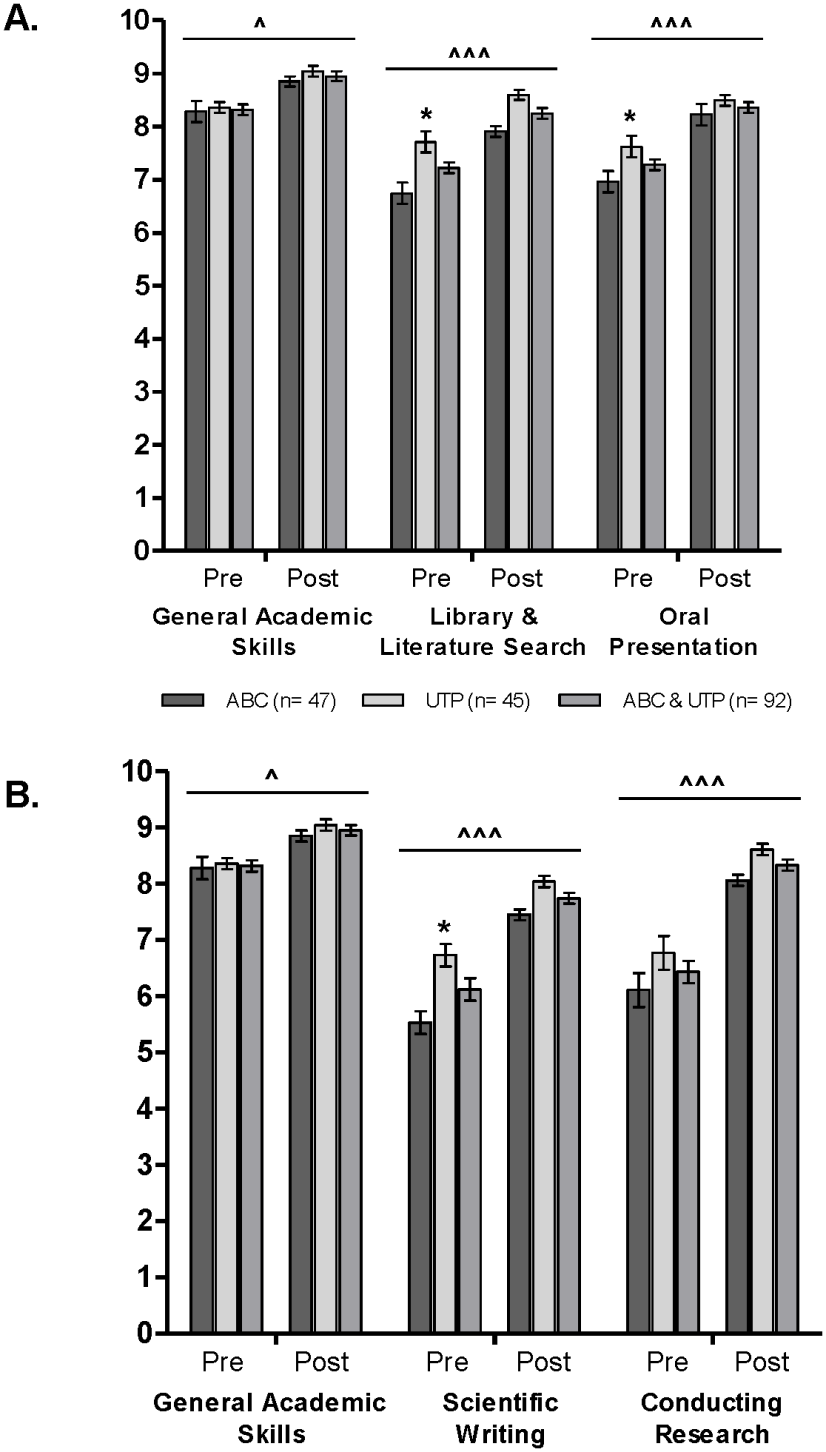
ABC and UTP participant self-assessment of research skills before and after the summer research experience (2006, 2010–2012). The ratings are presented as means ± SEM (scale: 1–10). The self-assessed general academic skill mean ratings are displayed alongside the self-assessed research skill mean ratings as a standard for comparison (A and B). *p<0.05 for the Pre mean rating comparison between the ABC and the UTP group. ∧*p*<0.05 for the Pre to Post paired mean rating comparison for all of the groups. ∧∧∧*p*<0.001 for the Pre to Post paired mean rating comparison for all of the groups.

The ABC and the UTP participants were asked to rate their perceived skill level (on a scale from 1 through 10) for the following 5 research skills: scientific writing, oral presentation, library and literature search, conducting research and general academic skills before and after the summer research experience. The participant self-ratings are shown in Figure 1 and are presented as means ± SEM. Except for general academic skills and conducting research, the UTP participant mean ratings tended to be higher than those of the ABC participants before the summer research experience, and these mean differences in scientific writing, oral presentation and library skills were statistically significant (*t*(90) = −3.12, *p* = 0.002, *t*(90) = −1.99, *p* = 0.049 and *t*(90) = −2.83, *p* = 0.006, respectively). The changes in scientific writing, oral presentation, library and literature search, and in conducting research were all large in magnitude (See Table 3) from PRE to POST for the ABC and the UTP groups and when both groups were combined. The greatest mean gains for both groups after the summer research experience were in “conducting research” (ABC η^2^, 0.42; UTP η^2^, 0.47) and in “scientific writing” (ABC η^2^, 0.45; UTP η^2^, 0.44).

**Table 3:** Effect Size Estimates^a^^,^ b for Gains in Research Skills and Self-Efficacy Outcomes After the Summer Research Experience, ABC & UTP Participants (2006, 2010–2012).

	ABC (*N* = 47)			UTP (*N* = 45)			ABC & UTP (*N* = 92)		
	PRE	POST		PRE	POST		PRE	POST	
	Mean ± SD	Mean ± SD	η^2^	Mean ± SD	Mean ± SD	η^2^	Mean ± SD	Mean ± SD	η^2^
Scientific Writing	5.53±1.9	7.45±1.2	0.45	6.73±1.7	8.04±0.9	0.44	6.12±1.9	7.74±1.1	0.44
Oral Presentation	6.96±1.6	8.23±1.3	0.43	7.62±1.5	8.49±0.8	0.29	7.28±1.6	8.36±1.1	0.36
Library & Literature Search	6.74±1.7	7.91±1.0	0.35	7.71±1.5	8.60±1.0	0.29	7.22±1.6	8.25±1.1	0.32
Conducting Research	6.11±2.1	8.06±1.1	0.42	6.77±2.1	8.61±0.8	0.47	6.43±2.1	8.33±1.0	0.44
General Academic Skills	8.28±1.4	8.85±1.1	0.10	8.36±1.1	9.04±0.7	0.34	8.32±1.3	8.95±0.9	0.17
Academic Self-Efficacy	43.96±6.61	45.21±5.47	0.06	43.87±6.09	46.69±4.92	0.19	43.91±6.32	45.93±5.23	0.12
Research Self-Efficacy	32.21±7.46	39.09±6.31	0.33	35.76±6.41	42.38±4.83	0.49	33.95±7.16	40.70±5.84	0.39

ABC = Apprenticeship Bridge to College, UTP = Undergraduate Training Program.

a The effect size reported is based on a GLM repeated measures model analyzed separately for each independent group [η^2^ = Sum of Squares (Effect)/Sum of Squares (Total)].

b The interpretation of the reported effect size was according to Cohen’s effect size magnitude criteria for the behavioral sciences (η^2^ = 0.01 small; 0.09 medium; 0.25 large) [44].

### Academic and Research Self-efficacy

**Figure 2:**
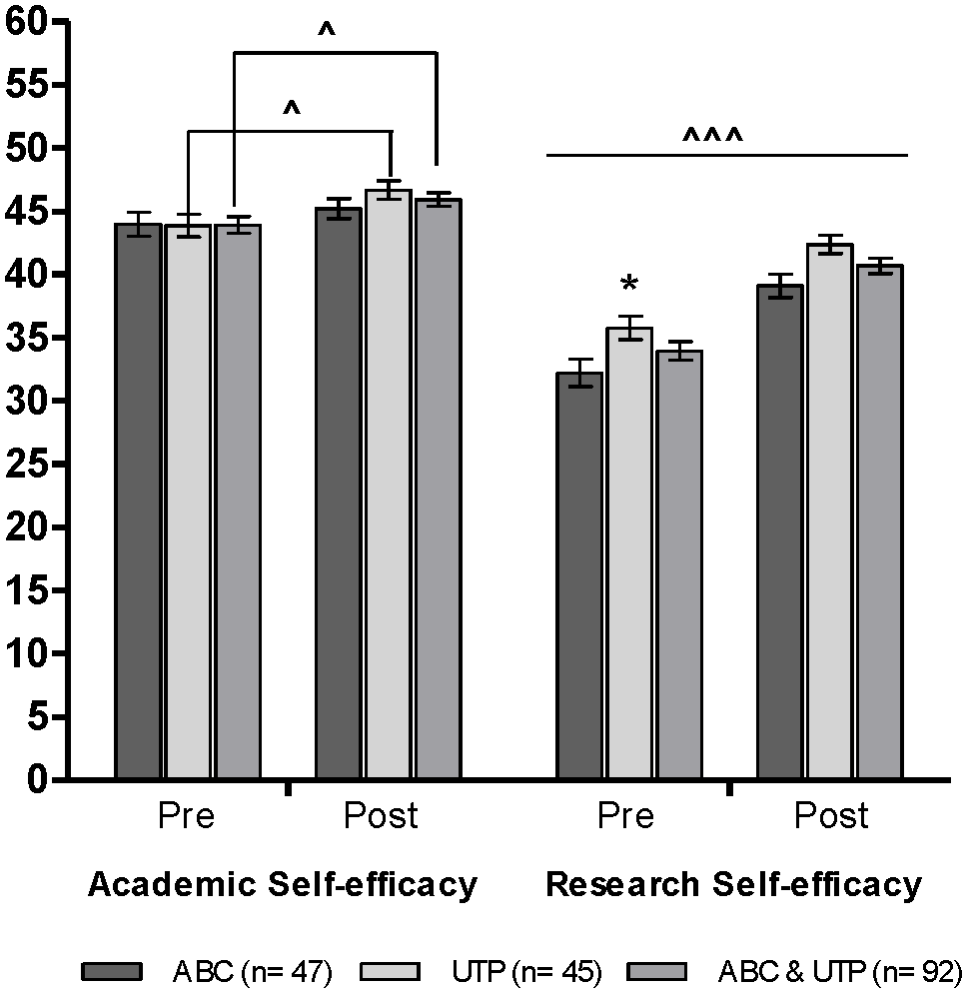
ABC & UTP participant self-assessment of academic and research self-efficacy before and after the summer research experience (2006, 2010–2012). The academic and research self-efficacy variables are presented as means ± SEM (maximum possible is 60). *p<0.05 for the Pre mean comparison between the ABC and the UTP group. ∧*p*<0.05 for the Pre to Post paired mean comparison for the indicated group. ∧∧∧*p*<0.001 for the Pre to Post paired mean comparison for all of the groups.

The academic and the research self-efficacy results for the participants before (PRE) and after (POST) the summer research experience are shown in Figure 2 and quantitatively in Table 3. Although the academic self-efficacy means were almost identical for the ABC and the UTP participants before and after the summer research experience, the UTP research self-efficacy means were significantly higher than the ABC research self-efficacy means at both time points (PRE *t*(90) = −2.44, *p* = 0.017 and POST *t*(90) = −2.80, *p* = 0.006, see Figure 2). Moderate gains in academic self-efficacy from baseline to post summer research experience were apparent for the UTP participant group (η^2^, 0.19) and for the combined group (η^2^, 0.12) but not for the ABC participant group alone (η^2^, 0.06, see Figure 2). However, both the ABC and the UTP participants reported large, statistically significant gains in research self-efficacy at the end of the summer research experience (ABC η^2^, 0.33; UTP η^2^, 0.49, see Table 3). At baseline, the participant academic and research self-efficacy ratings were correlated for the UTP participants (*r*(43) = 0.42, *p* = 0.004) but not for the ABC participants (*r*(45) = 0.27, *p* = 0.065). However, we observed a stronger measure of association between the two self-efficacy variables for both the UTP (*r*(43) = 0.45, *p* = 0.002) and the ABC (*r*(45) = 0.42, *p* = 0.003) participants after the summer research experience.

### Best Practices and Impact of the Summer Research Experience

Table 4 summarizes the most beneficial aspects of the summer research experience as identified by the ABC and the UTP participants on the exit surveys. The participant open-ended responses were coded for conceptual analysis through selective reduction and further categorized under the four sources of self-efficacy: mastery experiences, vicarious experiences, verbal persuasion, and physiological and affective states. None of the coded participant responses were conceptually representative of physiological and affective states; therefore, this self-efficacy source of influence is not listed in Table 4. The ABC and the UTP participants overwhelmingly identified mastery experiences as the most influential aspect of the summer research experience (74%). Roughly half of the participants identified the actual hands-on research experience as the best aspect of the summer research experience while the rest indicated the culminating research symposium and the overall learning experience as the most beneficial aspects. Vicarious experiences, in terms of the mentor experience and the influence of a high-achieving peer group, were the second most important aspect of the summer research experience, according to the participants. Table 5 summarizes the participant provided responses regarding the impact of the summer research experience on future goals with respect to school and/or career. We observed five broad themes when the participant responses were coded for content analysis: science career affirmation, increased science skills, increased confidence in science ability, increased motivation to pursue a science career, and increased time management and study skills. Around 80% of both the ABC and the UTP participants reported science-pipeline sustaining gains as a result of the summer research experience. About 58% reported affirmation for a science career, either for a new or previously held science career goal, while 14% reported increased confidence in science ability, and 8% reported increased motivation to pursue a science career.

**Table 4:** Summary List of the Most Valuable Aspects of the Summer Research Experience as Reported by the ABC & UTP Participants^a^ (2008, 2010–2012).

	ABC (*N* = 49)	UTP (*N* = 53)	ABC & UTP (*N* = 102)
Enactive Mastery Experiences^b^ (Total)	78% (38 of 49)	70% (37 of 53)	74% (75 of 102)
The hands-on research experience	53% (26 of 49)	51% (27 of 53)	52% (53 of 102)
The culminating research symposium	12% (6 of 49)	11% (6 of 53)	12% (12 of 102)
The overall learning experience	12% (6 of 49)	8% (4 of 53)	10% (10 of 102)
Vicarious Experiences (Modeling)^b^ (Total)	22% (11 of 49)	23% (12 of 53)	23% (23 of 109)
The mentor experience	18% (9 of 49)	13% (7 of 53)	16% (16 of 102)
The influence of ahigh-achieving peer group	4% (2 of 49)	9% (5 of 53)	7% (7 of 102)
Verbal Persuasion^b^ (Total)	-	8% (4 of 53)	4% (4 of 102)
The encouragement and the support	-	8% (4 ’of 53)	-

ABC = Apprenticeship Bridge to College, UTP = Undergraduate Training Program.

a Participant exit surveys were reviewed for content-analysis; participants were asked to provide details regarding the most valuable/most beneficial aspect of the summer research experience. Percentages may not add to one-hundred due to rounding.

b Bandura, A. (1997). Self-Efficacy: The Exercise of Control. New York, W.H. Freeman and Company. Four (4) sources of self-efficacy influence: Enactive Mastery Experiences, Vicarious Experiences (Modeling), Verbal Persuasion, and Physiological and Affective States. No ABC or UTP participant responses were representative of Physiological or Affective States.

**Table 5:** Summary List of the Impact of the Summer Research Experience on Future Goals as Reported by the ABC & UTP Participants^a^ (2008, 2010–2012).

	ABC (*N* = 50)	UTP (*N* = 46)	ABC & UTP (*N* = 96)
Science Career Affirmation (Total)	56% (28 of 50)	61% (28 of 46)	58% (56 of 96)
Affirmation for a biomedical clinical career	14% (7 of 50)	6% (3 of 46)	10% (10 of 96)
Affirmation for a biomedical clinicalcareer with an added research component	18% (9 of 50)	20% (9 of 46)	19% (18 of 96)
Affirmation for a biomedical research career	24% (12 of 50)	35% (16 of 46)	29% (28 of 96)
Increased Science Skills (Total)	22% (11 of 50)	9% (4 of 46)	16% (15 of 96)
Reading, Writing, Poster& Oral Presentation Skills	8% (4 of 50)	4% (2 of 46)	6% (6 of 96)
Science Knowledge & Lab Skills	14% (7 of 50)	4% (2 of 46)	9% (9 of 96)
Increased Confidence in Science Ability	10% (5 of 50)	17% (8 of 46)	14% (13 of 96)
Increased Motivation to Pursue a Science Career	6% (3 of 50)	11% (5 of 46)	8% (8 of 96)
Increased Time Management & Study Skills	6% (3 of 50)	2% (1 of 46)	4% (4 of 96)

ABC = Apprenticeship Bridge to College, UTP = Undergraduate Training Program.

a Participant exit surveys were reviewed for content-analysis; participants were asked about the impact of the summer research experience on future goals with respect to school and/or career. Percentages may not add to one-hundred due to rounding.

### STEM Educational Progress

The STEM educational pipeline progress of the ABC and the UTP participants is summarized in Table 6. Although a total of 132 high school students participated in the ABC program from 1997–2012, the follow-up data is for 116 participants. Loss to follow-up also occurred for college degree discipline data and after college graduation for some ABC participants (mainly from the 1997–1999 program years). Likewise, although 139 undergraduate students participated in the UTP program from 1997–2012, the follow-up data is for 136 participants.

**Table 6:** STEM Educational Progress for the ABC (1997–1999, 2001–2012) & the UTP (2001–2012) Participants^a^.

	ABC	UTP	ABC & UTP
Number of Participants with Follow-up Data	116	136	252
Currently Progressing Through High School	12% (14 of 116)	-	6% (14 of 252)
Graduated from High School	88% (102 of 116)	-	-
Currently Progressing Through College	49% (50 of 102)	21% (29 of 136)	33% (79 of 238)
Graduated from College	48% (49 of 102)	76% (103 of 136)	64% (152 of 238)
Graduated from College with a STEM degree*	67% (30 of 45)	90% (93 of 103)	83% (123 of 148)
Biochemistry	20% (6 of 30)	9% (8 of 93)	11% (14 of 123)
Biology	63% (19 of 30)	82% (76 of 93)	77% (95 of 123)
Biophysics	-	1% (1 of 93)	1% (1 of 123)
Biotechnology	3% (1 of 30)	1% (1 of 93)	2% (2 of 123)
Chemistry	3% (1 of 30)	5% (5 of 93)	5% (6 of 123)
Computer Science	3% (1 of 30)	-	1% (1 of 123)
Math	3% (1 of 30)	1% (1 of 93)	2% (2 of 123)
Nursing	3% (1 of 30)	1% (1 of 93)	2% (2 of 123)
Time-to-degree, STEM only, Years (Mean ± SD)	4.3±0.8	4.4±0.8	4.3±0.8
Enrolled in Graduate School*	55% (23 of 42)	78% (80 of 103)	71% (103 of 145)
Enrolled in LLU for Graduate Program*	61% (14 of 23)	43% (34 of 80)	47% (48 of 103)
Graduate Program*			
MD	48% (11 of 23)	44% (35 of 80)	45% (46 of 103)
MD/PhD	9% (2 of 23)	6% (5 of 80)	7% (7 of 103)
MD/MPH	4% (1 of 23)	-	1% (1 of 103)
PhD-Basic Science	13% (3 of 23)	15% (12 of 80)	15% (15 of 103)
DDS	4% (1 of 23)	5% (4 of 80)	5% (5 of 103)
PharmD	-	1% (1 of 80)	1% (1 of 103)
DPT	-	3% (2 of 80)	2% (2 of 103)
JD	-	1% (1 of 80)	1% (1 of 103)
Master of Arts	9% (2 of 23)	1% (1 of 80)	3% (3 of 103)
Master of Business Administration	-	1% (1 of 80)	1% (1 of 103)
Master of Public Health	9% (2 of 23)	13% (10 of 80)	12% (12 of 103)
Master of Science	4% (1 of 23)	8% (6 of 80)	7% (7 of 103)
Master of Social Work	-	3% (2 of 80)	2% (2 of 103)

STEM = Science, Technology, Engineering, Math, ABC = Apprenticeship Bridge to College, UTP = Undergraduate Training Program, LLU = Loma Linda University.

a Participant progress as of January 2013.

*Percentages were calculated based on valid follow-up data (excluding cases that were lost to follow-up) and may not add to one-hundred due to rounding.

The educational pipeline progress of the participants is presented in a cross-sectional manner and current as of January, 2013. Enrollment in a graduate program also includes those who may have already completed their graduate degree at this time point. Of the ABC participants with college degree follow-up data, 67% graduated from college with a STEM degree, mainly with biology and biochemistry degrees. Out of the UTP participants that have already graduated from college, 90% graduated with a STEM degree, with biology and biochemistry degrees also predominating. About 55% of ABC participants with college graduation and graduate enrollment data enrolled in a graduate degree program, and of those, 61% enrolled in LLU for a graduate program. For UTP participants, 78% of all those who graduated from college enrolled in a graduate degree program, and 43% enrolled in LLU for their respective graduate program. When the ABC and UTP outcomes are combined, about 45% of those who enrolled in graduate education pursued MD degrees, and another 23% pursued basic science PhD’s or combined doctoral degrees (MD/PhD and MD/MPH). Additionally, about 19% of the combined group not enrolled in a doctoral program enrolled in a Master’s of Public Health degree program or a Master’s of Science (STEM discipline) degree program.

## Discussion

### Impact of the ABC and UTP Programs on Diversity and Inclusion at LLU

There is a dire need for graduate and professional schools to establish evidence-based pipeline programs that will increase the student diversity of their STEM doctoral degree programs. An untapped yet promising pool of students are capable high school students that have a strong STEM interest but may lack the skills and the guided mentoring needed to succeed in competitive STEM disciplines [12]. As of 2014, 403 research internships have been awarded to 158 Apprenticeship Bridge to College (ABC) high school participants and 162 Undergraduate Training Program (UTP) college participants. Of the 271 participants included in this study, 51 students have participated in this summer research internship program more than once, and 16 have participated in both the UTP and the ABC program. The number of multiple-summer participants may convey participant satisfaction with the summer research internship and is evidence that the participants see the benefits of continued training in our research environment. Through partnerships with local high schools and minority-serving institutions, the LLU summer research pipeline program has filled an important gap in building research capacity and in exposing students to our graduate school. Collectively, from both programs, 47% (48 of 103) of those who enrolled in graduate education have enrolled at LLU. From the ABC program alone, 61% (14 of 23) of those who enrolled in graduate education have enrolled at LLU; thus, we have reason to believe that the research internship is contributing to the diversity and inclusion mission of our institution.

### Participant-assessed Changes in Research Intent, Skills and Self-Efficacy

While most of the published literature has focused on assessing the benefits of a research internship overall and mainly through retrospective analysis, we present the changes in research intent, research skills, and research self-efficacy in a subgroup of ABC and UTP participants with valid paired data. This method of evaluation accounts for the inherent selection bias in a research internship that uses a non-random participant selection process. By using the participants’ baseline values as their own controls and focusing on the differences from the baseline values, it is possible to more accurately determine the changes and benefits of the research internship in a group of students with prior STEM interest. One of the driving questions underlying our evaluation was whether participation in the research internship would increase the likelihood that the participants would choose future research endeavors. We hypothesized that a positive research experience would familiarize participants with the process of hands-on biomedical research and would lead them to incorporate research into their future careers. Table 2 provides convincing evidence that both the ABC and the UTP participants were more likely to consider incorporating research into their future careers after the summer research internship. This effect is more pronounced for the ABC participants who, as a whole, varied more in their baseline terminal degree intent and were less likely to consider incorporating research into their career before the summer research experience. Although this effect could be due to the ABC participants’ prior lack of knowledge regarding the day-to-day details that actual research entails, it may also point out that ABC students with a STEM interest may be more influenced by an early hands-on research experience than their UTP college student counterparts and may be more likely to choose a research career as a result of this early exposure [22]. That both the ABC and the UTP groups shifted in intent from mainly MD (55%) to MD/PhD (44%) degrees after the summer research experience also demonstrates that the internship helps to shape intent for research-intensive doctoral degrees, at least immediately after the SRE. While this change in intent is dramatic, other factors besides intent may play a more prominent role in terminal degree pursuit and may result in a lower proportion of students actually completing an MD/PhD [23]; this is also apparent in the Table 6 enrollment data.

According to the social cognitive theory, shifts in career selection processes may stem from changes in self-efficacy beliefs [20]. Because motivation, affective states and actions are based more on what people believe than on what is objectively true, we asked the ABC and the UTP participants to self-rate their ability on a variety of research skills before and after the summer research experience. Overall, except for General Academic Skills, the ABC participant-ratings were lower than the UTP participant-ratings. We observed this same trend in the baseline academic and research self-efficacy values; the academic self-efficacy means were not statistically different between the two groups whereas the research self-efficacy means were statistically higher for the UTP participants at both time points. These findings are not surprising given that the ABC students were not science majors and had limited laboratory experience prior to participation in the research internship.

Despite the baseline differences, the effect of the research internship was comparable across both groups; that is, both groups reported large, statistically significant gains in research skills and research self-efficacy as a result of the summer research experience. These results have two important and related implications: high school students are not limited in ability to perform hands-on research simply because they lack college level lab course experience *and* college level didactic course prerequisites are not required for high school students to successfully reap the benefits of a guided research experience. Unlike various high school programs reported in the literature, the LLU ABC research internship is not formatted as an introductory didactic science course. It does not have a remediation, academic or college preparatory component to it, nor does it use a gradual approach to research that is characterized by teaching a single lab technique to all students [15]–[18], [24]. Rather, the ABC students are challenged by working on a mentored, individualized, hands-on research project comparable to the research experience of the UTP college students. Based on exit surveys, this intensive immersion is critical for the ABC participants because it provides the students with a real taste for research and allows them to rise to the challenge of making an actual contribution to the project they join.

Consistent with the self-efficacy theory, we found that academic and research-efficacy were distinct domains and were poorly correlated for the ABC participants before the summer research experience. While theoretically intuitive, this finding distinguishes perceived academic self-efficacy from perceived research self-efficacy in a group of capable high school students and shows that STEM interest and academic confidence alone will not immediately correlate to confidence in science process skills. Proficiency in science process skills (such as identifying variables, recognizing operational definitions, data and graph interpretation, experimental design, and identifying hypotheses) has been shown to improve performance in rigorous entry-level biology courses [14]. For the UTP participants, most of whom were STEM majors at the time of participation, research efficacy was weakly but significantly correlated with academic efficacy before the summer research experience. Although the participants reported moderate to large gains in “General Academic Skills” and small to moderate gains in academic self-efficacy, the largest gains were reported for “Conducting Research,” “Scientific Writing,” and research self-efficacy. For the ABC participants, academic self-efficacy remained relatively constant from PRE to POST (See Figure 2) indicating that the research internship mainly targeted the research capability and the STEM confidence of these participants.

Changes in perceived research-efficacy have important implications for both the ABC and the UTP participants. For the UTP participants, the benefits of improved research abilities and increased efficacy are likely to affect subsequent performance in science courses [25] as well as positively influence the self-assurance with which UTP students approach graduate education [20], [26]–[28]. A resilient sense of efficacy will also likely contribute to STEM course success for the ABC participants, but, more importantly, it may commit the high school students, early-on, to a STEM research career over a clinical or professional career [22], [29], [30]. Students who have participated in high school summer research experiences may be more likely to pursue structured research activities as undergraduates, indicating that the selection processes following a high school summer research experience will likely lead to an even stronger commitment for STEM disciplines and terminal degree goals [22], [30], [31]. This is supported by the fact that, at the end of the summer research experience, the estimated odds of choosing “very likely” to pursue a biomedical research doctoral degree improved by about 35% for every five unit increase in research self-efficacy [OR 1.35 (95% CI 1.10, 1.65), *p* = 0.004] for the combined group (*N* = 90).

### Participant-identified “Best Practices” and the SRE Impact on Future Goals

Free-format narrative responses and interviews have previously been effective in identifying the utility and the broad gains of undergraduate research experiences [32]–[38]. In terms of frequency, the two most important components of the research experience were, as identified by the participants, the hands-on research experience (53 of 102) and the mentor experience (16 of 102). When further classified according to source of influence, enactive mastery experiences and vicarious experiences were the two most important sources of self-efficacy influence. The fact that the participants did not simply identify the acquisition of lab skills as the most beneficial aspect of the summer research experience supports the idea that self-efficacy is not primarily affected by perceived skills but rather is changed through a complex integration of efficacy influences. Although the different forms of efficacy influence rarely operate independently from each other, the frequency with which the participants identified mastery experiences, or the actual hands-on research experience, as the most important aspect of the program supports the social cognitive claim that enactive mastery experiences are the most influential source of self-efficacy information because they provide the most authentic evidence that one has what it takes to succeed [20]. Mastery of the difficult task of research likely resulted in raising the research-efficacy beliefs of both the ABC and the UTP participants (See Figure 2). Vicarious experiences, or social modeling, also likely contributed to the participant perceived changes in research efficacy. In their mentors and in their peer group, the participants observed other individuals similar to themselves performing successfully and lending credibility to the belief that they too have the capability to master comparable activities. The one-to-one mentor experience most likely conveyed effective coping strategies and better ways of doing things, especially for the young inexperienced researchers [20]. It is important to note that for a small number of participants, it was a combination of two of the listed elements that proved to be the most beneficial. While it may be worthwhile to explore how the different combinations of components contributed to the participant perceived success, we simply did not have a large enough sample of multiple-component responses to examine this effect.

Self-efficacy beliefs are not an end in themselves. Within the social cognitive framework, efficacy beliefs have diverse effects on cognitive, motivational, affective and selection processes [19], [20]. When the ABC and the UTP participants were asked about the impact of the research internship on future goals with respect to school or career, 80% reported science-pipeline sustaining gains consistent with what has previously been reported in the literature [25], [29], [35], [36], [39], [40]. In terms of social cognitive processes, about 58% (56 of 96) of the participants indicated that the internship increased affirmation for a science career, a likely selection process response. Similarly, the participant-reported increases in motivation and science confidence may indicate changes in motivational processes. We noted that the ABC participants were proportionally more likely to report gains in science skills, time management and study skills when compared to the UTP participants even though the research internship is not structured as a college preparatory or study skill forming experience for either group. Rather, this finding is consistent with the social cognitive tenet that maintains that cognitive processes are especially influential in the early and intermediate phases of competency development [20]. That is, the ABC students may have been more likely to project the benefits of the research experience into college or science course success.

### Underrepresented Minority STEM Pipeline Progress

The follow-up data for our Summer Health Disparities Research Program indicates that the participants are successfully persisting through the STEM academic pipeline. While this study is constrained to comparing the impact of the SRE in terms of gains in research skills and research self-efficacy, we believe it is important to report the progression of the participants through the STEM academic pipeline especially as it pertains to the number of students who return to our institution for graduate level education. This data can also be of value in future studies on this cohort of students.

The longitudinal results for the ABC participants who graduated from college show that 67% graduated with a STEM degree in an average of 4 years. The ABC participants chose mainly biology and biochemistry majors that were consistent with their application interests and with the likely influence of a biomedical research internship. Since 90% of the UTP students were majoring in a STEM discipline at the time of participation in the program, the 90% STEM degree completion rate in an average of 4 years represents their success in STEM disciplines, especially for those who participated in the research internship after their freshman or sophomore year in college. The number of pipeline college graduates who enrolled in a graduate program (71%) is comparable to or higher than what has previously been reported by other groups [25], [26], [34], [41]. Ultimately, we believe that our pipeline program is fulfilling its mission to train and build research capacity in the next generation of URM biomedical leaders given that 75% (77 of 103) of those students in graduate programs are enrolled in biomedical research or professional doctoral-level programs.

### Limitations and Future Studies

This study has several limitations. We cannot completely account for the selection bias that may be prominent in a research internship program that has both eligibility and selection criteria. We address this issue, however, by presenting quantitative and qualitative analyses of paired data. By using the participants as their own controls and by focusing on the changes from their baseline values, we not only dismiss as many of these extraneous sources of variation as possible but also account for the effect of participating in a research internship once. Because the participants may still be different in motivational or academic capacity, we present the participant self-assessed research skill mean ratings and the research-efficacy mean ratings alongside the self-assessed general academic skill mean ratings and the academic-efficacy mean ratings as standards for comparison. We point out that self-efficacy is widely believed to be a domain specific, or situation specific, competence belief [20]; therefore, it is appropriate to measure research efficacy independently of academic efficacy in a research internship intervention.

A particular strength of our study is that we compare the high school participant results to the college participant results to demonstrate that the beneficial effect of our summer research experience is comparable across both groups regardless of the age or educational differences between the two groups. Although the results from the paired-data analyses represent a sub-sample of the overall participant group, we are confident that these results are representative of the larger sample because the main components of research internship (i.e., the mentored hands-on research experience) have not changed since the beginning of the program. We are currently continuing our efforts to follow-up our participants and will administer a formal online survey to gauge any other factors or experiences that may have added to their career choices and determination.

## Conclusion

At the time of the summer research experience the ABC participants were different than the UTP participants in two crucial ways: unlike the undergraduates, the high school students had not yet cleared the hurdle of STEM college gateway courses or faced the complex acquisition of science identity. While an adequate discussion of self-efficacy, role identity and value endorsement is outside the scope of this study, the work of Chemers, Estrada-Hollenbeck and Schultz sheds light on the foundational role of self-efficacy in the long-term commitment to a science career [42]–[44]. According to Chemers, participation in a research experience increases science self-efficacy which in turn enhances science identity and, consequently, leads to a stronger commitment to a STEM career [42]. The critical development of science self-efficacy as it influences the development of deeper measures of integration is supported by Estrada-Hollenbeck’s work that found that while self-efficacy was related to identity and values, the relative influence of each on long-term STEM career commitment may be mediated by the progression of the student through the academic pipeline [43].

For students aspiring towards a STEM career, the time at which science self-efficacy must be developed seems to be of pivotal importance. The positive benefits of undergraduate research experiences have previously been demonstrated; undergraduate students have widely regarded their research experience as beneficial [29], [39], [45] and have credited the research experience for developing self-confidence, analytical skills, independence, and motivation to learn [29], [31], [45]. Research experiences have also been linked to improved STEM course persistence and improved college graduation rates and GPA outcomes [26], [32], [33], [36], and have provided career path clarification for those with and without prior research interest [27], [29], [31], [34], [39]. The results of our study demonstrate many of those same STEM-pipeline sustaining gains except that they are reported by high school students. The work of Hurtado suggests that an early, structured research experience may catalyze the initial domain identity development needed for persistence in a STEM major [38], [46]. A pre-college research experience may also increase interest in pursuing a scientific research career [22] as well as increase the likelihood of participating in a health science research opportunity as a first-year college student [30]. Arguably, if underrepresented minority students [47], or all students for that matter [6], are less prepared to be science majors, the active learning experience of a summer research internship will not only raise the science literacy of the high school participants [48] but may also improve their performance in college level gateway classes [49]. If timing is of utmost importance, we propose that inquiry-based learning should begin prior to the freshmen year in college where the gains in research skills and research self-efficacy will likely reap the most benefits for the students, the STEM educational system, and the nation as a whole.
